# Prediction of trehalose-metabolic pathway and comparative analysis of KEGG, MetaCyc, and RAST databases based on complete genome of *Variovorax* sp. PAMC28711

**DOI:** 10.1186/s12863-021-01020-y

**Published:** 2022-01-06

**Authors:** Prasansah Shrestha, Min-Su Kim, Ermal Elbasani, Jeong-Dong Kim, Tae-Jin Oh

**Affiliations:** 1 Department of Life Science and Biochemical Engineering, Graduate School, Sun Moon University, Asan, 31460 Korea; 2 Department of Computer Science and Engineering, Sun Moon University, Asan, 31460 Korea; 3 Genome-based BioIT Convergence Institute, Asan, 31460 Korea; 4 Department of Pharmaceutical Engineering and Biotechnology, Sun Moon University, Asan, 31460 Korea

**Keywords:** KEGG, MetaCyc, RAST annotation, Trehalose metabolism, *Variovorax* sp. PAMC28711

## Abstract

**Background:**

Metabolism including anabolism and catabolism is a prerequisite phenomenon for all living organisms. Anabolism refers to the synthesis of the entire compound needed by a species. Catabolism refers to the breakdown of molecules to obtain energy. Many metabolic pathways are undisclosed and many organism-specific enzymes involved in metabolism are misplaced. When predicting a specific metabolic pathway of a microorganism, the first and foremost steps is to explore available online databases. Among many online databases, KEGG and MetaCyc pathway databases were used to deduce trehalose metabolic network for bacteria *Variovorax* sp. PAMC28711. Trehalose, a disaccharide, is used by the microorganism as an alternative carbon source.

**Results:**

While using KEGG and MetaCyc databases, we found that the KEGG pathway database had one missing enzyme (maltooligosyl-trehalose synthase, EC 5.4.99.15). The MetaCyc pathway database also had some enzymes. However, when we used RAST to annotate the entire genome of *Variovorax* sp. PAMC28711, we found that all enzymes that were missing in KEGG and MetaCyc databases were involved in the trehalose metabolic pathway.

**Conclusions:**

Findings of this study shed light on bioinformatics tools and raise awareness among researchers about the importance of conducting detailed investigation before proceeding with any further work. While such comparison for databases such as KEGG and MetaCyc has been done before, it has never been done with a specific microbial pathway. Such studies are useful for future improvement of bioinformatics tools to reduce limitations.

## Background

Metabolism refers to all biochemical processes that occur during the growth of a cell or an organism. Microbial metabolism involves a group of complex chemical compounds. It includes anabolism and catabolism for microorganisms to obtain energy and nutrients for survival and reproduction. A microbe’s metabolic properties are the foremost important factors in determining its condition. They may be accustomed to monitor biogeochemical cycles and industrial processes [1]. Therefore, the study of microbial metabolism is important. It has been a driving force for the growth and conservation of the planet’s biosphere [2]. In microorganisms, various metabolism pathways are involved [3]. *Variovorax* sp. PAMC28711 selected in this study to explore trehalose metabolism is one of cold adapted lichen-associated bacteria isolated from Antarctica. Analysis of enzymes from cold-adapted microorganisms has become common in recent years because cold-adapted enzymes from organisms living in Polar regions, deep oceans, and high altitudes have various benefits [4]. Genus *Variovorax* is a cold adapted, Gram-negative, motile bacterium that comes in a variety of shapes, including flat, slightly curved, and rod shapes. Because of the presence of carotenoid pigments, *Variovorax* colonies are yellow, slimy, and shiny [5]. There are several carbohydrate metabolism pathways in *Variovorax* sp. PAMC28711. One of them is trehalose metabolic pathway. Trehalose is a naturally occurring alpha-linked disaccharide formed by two molecules of glucose. It was first isolated by French chemist Marchellin Berthelot in the mid-nineteenth century from *Trehala manna,* a sweet substance obtained from nests and cocoons of the Syrian coleopterous insects (*Larinus maculatus* and *Larinus nidificans*) known to feed on the foliage of a variety of thistles. Trehalose is used for biopharmaceutical preservation of labile protein drugs and cryopreservation of human cells. It is also widely used in the food industry [6]. Trehalose can be used as an alternative carbon source in microorganisms [7]. There have been a lot of research studies about its biological and chemical properties as well as its use in living organisms [8]. Metabolic pathways can be predicted using a variety of online methods. Kyoto Encyclopedia of Genes and Genomes (KEGG) and MetaCyc are two well-known online databases that can be used to predict metabolic pathways. Genomes, biological processes, disorders, medications, and chemical compounds are all included in the KEGG database. KEGG can be used for bioinformatics research and education in genomics, metagenomics, metabolomics, and other omics studies, modeling and simulation in systems biology, and translational research in drug development [9]. MetaCyc is another pathway database. It is one of the most extensive databases of metabolic pathways and enzymes. Information in this database has been hand-curated from scientific literature. It covers every aspect of life, including chemical compounds, reactions, metabolic processes, and enzymes. Over 58,000 journals were used to compile this database [10, 11]. Rapid Annotation using Subsystem Technology (RAST) annotation engine was developed in 2008 to annotate bacterial and archaeal genomes. It functions by supplying a standard software pipeline for identifying and annotating genomic features such as protein-coding genes and RNA [12]. RAST and other annotation engines are pipelines that combine tools for detection and annotation of complex genomic features [13–16].

KEGG and MetaCyc are two well-known and popular databases for metabolic pathway prediction. To study trehalose metabolic pathway in *Variovorax* sp. PAMC28711 and predict enzymes involved in this pathway, these two databases were chosen in study. This is the first study to compare cold-adapted bacteria to well-known databases and predict missing enzymes using RAST annotation software for further analysis of results obtained from KEGG and MetaCyc databases. Furthermore, this paper provides insight into how to validate computational data’s outcomes and proceed further.

## Materials and methods

### Data sources

A complete genome information of *Variovorax* sp. PAMC28711 was obtained from the National Center for Biotechnology Information (NCBI) genome database (https://www.ncbi.nlm.nih.gov/) for this metabolic pathway study. The GenBank accession number of *Variovorax* sp. PAMC28711 is NZ_CP014517.1.

### Trehalose metabolic pathway prediction in *Variovorax* sp. PAMC28711 using bioinformatics tools

The KEGG pathway database (http://www.kegg.jp/ or http://www.genome.jp/kegg) and MetaCyc database (MetaCyc.org) were used to predict trehalose metabolic pathway in the complete genome of *Variovorax* sp. PAMC28711. During prediction of pathway via the annotated file, bioinformatics tools such as RAST annotation server (https://rast.nmpdr.org/rast.cgi) were used to find the missing enzyme.

## Results

### Comparison of programs for trehalose metabolic pathway in *Variovorax* sp. PAMC28711

The comparison of three programs (KEGG, MetaCyc, and RAST annotation) for the prediction of enzymes involved in trehalose metabolism in *Variovorax* sp. PAMC28711 is shown in Table 1. According to KEGG, *Variovorax* sp. PAMC28711 possessed only OtsA-OtsB and TreS pathways. MetaCyc database showed similar outcomes as KEGG database. The OtsA-OtsB pathway has two enzymes, trehalose-6-phosphate synthase (OtsA) and trehalose-6-phosphate phosphatase (OtsB). The TreS reversible pathway has one enzyme, trehalose synthase.

**Table 1:** Prediction of enzymes involved in trehalose metabolic pathway in *Variovorax* sp. PAMC28711

Program	Trehalose biosynthesis pathway					
	OtsA-OtsB		TreY-TreZ		TreS	Enzyme missing
	EC 2.4.1.15	EC 3.1.3.12	EC 5.4.99.15	EC 3.2.1.141	EC 5.4.99.16	
**KEGG**	O	O	X	O	O	EC 5.4.99.15
**MetaCyc**	O	O	X	O	O	EC 5.4.99.15
**RAST**	O	O	O	O	O	No

“O” represents the presence of the respective pathway and “X” represents the absence of the respective pathway

As shown in Table 2, MetaCyc version 22.5 (August 2018) had 2,688 pathways and KEGG version 87.0 had 339 metabolic modules (August 2018). In comparison to 530 maps found in KEGG, MetaCyc version 22.5 had 381 super pathways. KEGG version 87.0 had 11,004 reactions, while MetaCyc version 22.5 had 15,329. Super pathways and maps are useful for displaying how individual pathways interact and the broader biochemical context in which a pathway works. MetaCyc pathways can be viewed at various levels of details, including chemical structures for substrates. Furthermore, all MetaCyc pathway diagrams provide chemical and enzyme names, while KEGG module diagrams only provide incomprehensible identifiers [17].

**Table 2:** Comparison of MetaCyc/BioCyc and KEGG pathway databases

Category	MetaCyc (Base)	KEGG (Module)	MetaCyc (Superpathways)	KEGG (Map)
Pathway count	2,688	339	381	530
Pathway reactions	15,329	11,004	-	-

### Predicted trehalose metabolism pathways by KEGG and MetaCyc

**Fig. 1:**
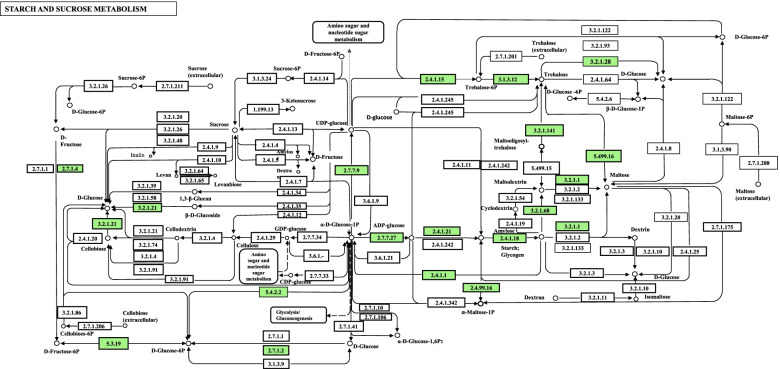
Snapshot of KEGG pathway map (vaa00500) “Starch and sucrose metabolism-*Variovorax* sp. PAMC28711 highlighted in red

Figure 1 shows trehalose metabolic pathway of *Variovorax* sp. PAMC28711 obtained from the KEGG pathway database [18–20]. Trehalose metabolism pathway comes under results of starch and sucrose metabolic pathway. Green boxes are hyperlinked to genes entries by converting K numbers (KO identifiers) to gene identifiers in their reference pathway, indicating the presence of genes in the genome and the completeness of the pathway. White boxes show missing enzymes in TreY/TreZ maltooligosyl-trehalose synthase (TreY)/maltooligosyl-trehalose trehalohydrolase (TreZ) pathway in the trehalose metabolic pathway. According to the KEGG pathway, *Variovorax* sp. PAMC28711 lacks enzyme maltooligosyl-trehalose synthase (TreY: EC 5.4.99.15), which makes the TreY/TreZ pathway incomplete.

**Fig. 2:**
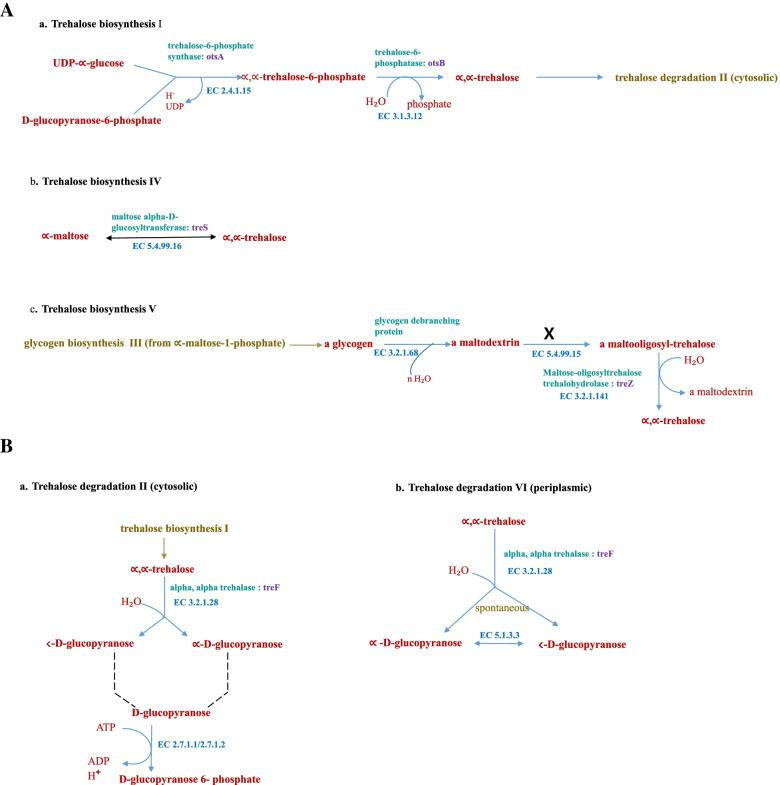
Trehalose metabolic pathway obtained from MetaCyc. **A** Trehalose biosynthesis pathway in *Variovorax* sp. PAMC28711. **B** Trehalose degradation pathway in *Variovorax* sp. PAMC28711. Note: “X” represents that the absence of the respective enzyme. Note: dashed line (without arrowheads) between two compound names implies that the two names are just different instantiations of the same compound. i.e., one is a specific name and the other is a general name, or they may both represent the same compound in different stages of a polymerization-type pathway. If the enzyme is shown in bold, there is experimental evidence for this enzymatic activity

Figure 2 shows results of trehalose biosynthesis and degradation pathways in *Variovorax* sp. PAMC28711 obtained from the MetaCyc database. Figure 2A (a, b, and c) shows three trehalose biosynthesis pathways in *Variovorax* sp. PAMC28711: trehalose biosynthesis I (OtsA: EC 2.4.1.15 and OtsB: EC 3.1.3.12), trehalose biosynthesis IV (TS: EC 5.499.16), and trehalose biosynthesis V (TreX: EC 3.2.1.68, TreY: EC 5.4.99.15, and TreZ: EC 3.2.1.141). According to MetaCyc, trehalose biosynthesis V has three enzymes (TreX: EC 3.2.1.68, TreY: EC 5.4.99.15, and TreZ: EC 3.2.1.141). However, *Variovorax* sp. PAMC28711 lacks enzyme TreY: EC 5.4.99.15, which prevents the trehalose biosynthesis V pathway from being complete. Therefore, it is assumed that the trehalose biosynthesis V pathway is absent in *Variovorax* sp. PAMC28711 as results suggest that only two trehalose biosynthesis pathways are involved in this strain.

### Trehalose metabolic pathway in *Variovorax* sp. PAMC28711

**Fig. 3:**
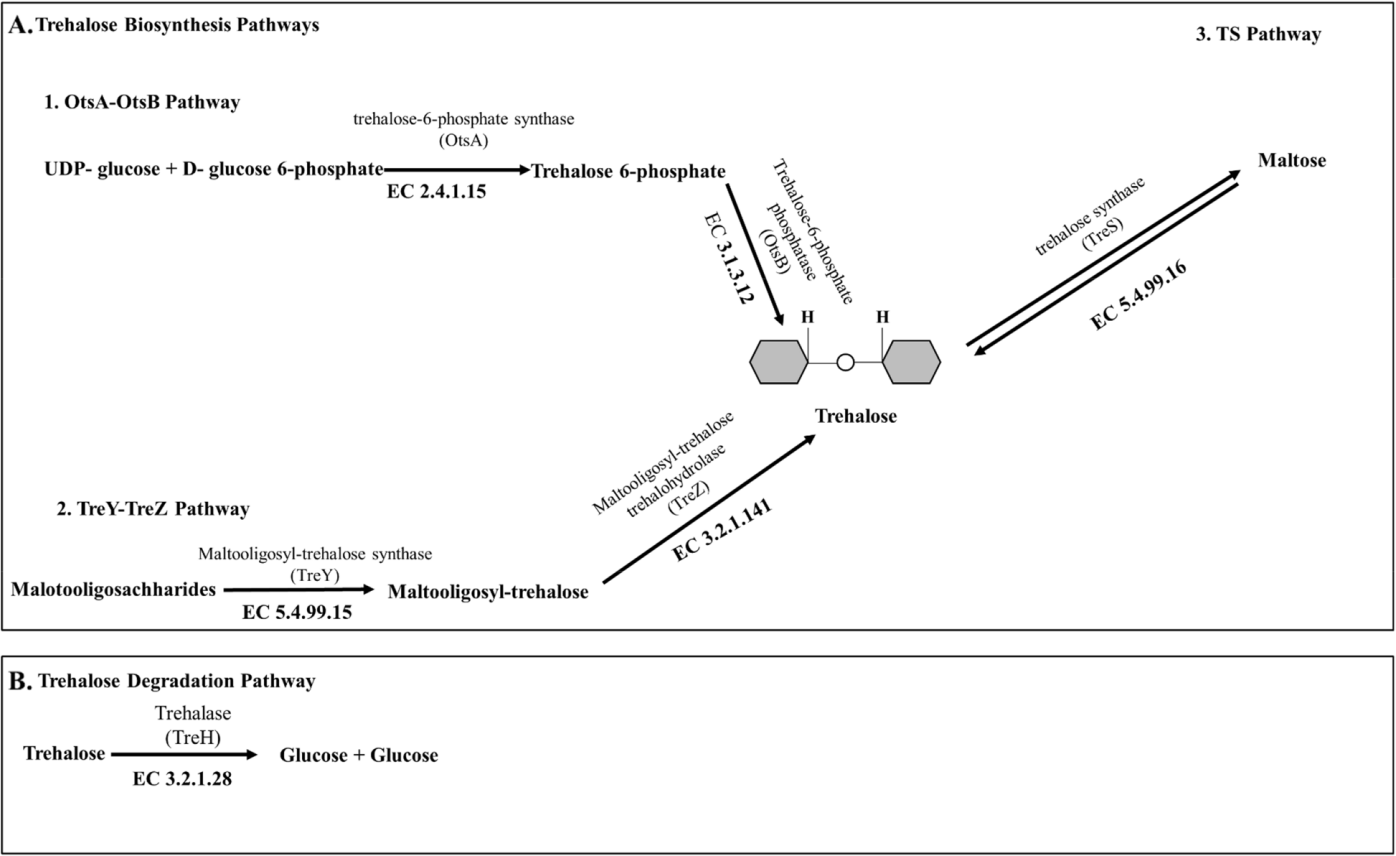
Complete trehalose biosynthetic pathway (**A**) and degradation pathway (**B**) in *Variovorax* sp. PAMC28711

**Fig. 4:**
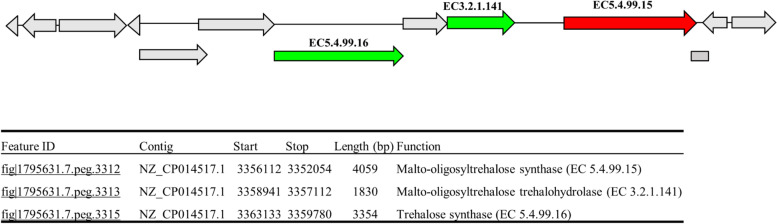
Graphical representation from RAST annotation database for trehalose biosynthesis genes in *Variovorax* sp. PAMC28711

*Variovorax* sp. PAMC28711 has three pathways for trehalose biosynthesis OtsA/OtsB, TS, and TreY/TreZ. Enzymes involved in these three pathways are trehalose 6-phosphate synthase (OtsA: EC 2.4.1.15), trehalose 6-phosphate phosphatase (OtsB: EC 3.1.3.12), trehalose synthase (TS: EC 5.499.16), maltooligosyl-trehalose synthase (TreY: EC 5.4.99.15), and maltooligosyl-trehalose trehalohydrolase (TreZ: EC 5.3.2.1.141). The trehalose degradation pathway (TreH) in *Variovorax* sp. PAMC28711 possesses one enzyme, trehalase. Figure 3 summarizes the overall trehalose metabolic pathway in *Variovorax* sp. PAMC28711. The missing enzyme (TreY: EC 5.4.99.15) was found from results of RAST annotation through SEED Viewer which started and stopped at 335612 to 3352054 coding sequence (CDS) (Fig. 4). Therefore, the three biosynthesis pathways of *Variovorax* sp. PAMC28711 are complete.

## Discussion

Trehalose metabolism is one of metabolism pathways for carbohydrates. Five distinct pathways for trehalose synthesis have been described. However, there is only one pathway for trehalose synthesis in fungi, plants, and animals [21]. These five distinct pathways are: TreY/TreZ (EC 5.4.99.15/EC 3.2.1.141) pathway (present in archaea and bacteria), TreS (EC 5.499.16) pathway (present only in bacteria), OtsA/OtsB (EC 2.4.1.15/EC 3.1.3.12) pathway (present in archaea; bacteria; fungi; plants; arthropods; and protists), TreP (EC 2.4.1.64) pathway (present in prostists, bacteria, and fungi), and TreT (EC 2.4.1.245) pathway (present in archaea and bacteria) [22]. Trehalose biosynthesis in bacteria has three pathways: OtsA/B, TreY/Z, and TreS [23]. However, according to KEGG results for trehalose metabolism in *Variovorax* sp. PAMC28711, there are only two trehalose biosynthesis pathways: the OtsA/B pathway and the TreS pathway. We used RAST annotation server to find the missing enzyme, maltooligosyl-trehalose synthase (TreY: EC 5.4.99.15), in KEGG results. RAST annotation is an excellent starting point for a more systematic annotation initiative since it can differentiate between two types of annotation and use reasonably accurate subsystem-based statements as the basis for a through metabolic reconstruction [24]. As a result, we discovered that the enzyme we were looking for was present (TreY: EC 5.4.99.15) in the RAST annotation database. It was fascinating to discover that *Variovorax* sp. PAMC28711 used all three trehalose biosynthesis pathways. In addition, we examined MetaCyc pathway database to compare our results and found that the enzyme maltooligosyl-trehalose synthase (TreY: EC 5.4.99.15) was also missing in this database (Table 1, Figs. 1, and 2A). TreY (maltooligosyl-trehalose synthase) is also known trehalose biosynthesis V. The basic method for determining whether a pathway occurs in an organism is based on the existence of the pathway’s enzymes in that organism (usually deduced by the presence of genes predicted to encode such enzymes in the annotated genome). When some enzymes are not detected in a database, it might be because some enzymes are not correctly recognized or annotated due to limited knowledge, variances, and sequences that could not meet the defined arbitrary threshold of two databases [25]. It might also because some pathways have overlapping parts, making it difficult to identify the enzymes involved. RAST can achieve precision, quality, and completeness is because it is based on the use of a growing library of manually curated subsystems as well as protein families derived largely from subsystems (*FIGfams*) [26]. The KEGG pathway database is a series of KEGG pathway maps, which are hand-drawn graphical diagrams that describe molecular pathways in metabolism, genetic information processing, environmental information processing, cellular processes, organismal systems, human diseases, and drug production [27]. A five-digit number preceded by one identifies each pathway: map, ko, ec, rn, and three- or four-letter organism code. The pathway map is drawn and updated with the notation [27]. Other maps with coloring are all computationally generated. KEGG pathway maps are based on experimental evidence of specific species. They are intended to be applicable to other organisms as well since different organisms, such as humans and mice, often share similar pathways made up of functionally identical genes known as orthologous genes or orthologs [28]. MetaCyc is a curated database of experimentally elucidated metabolic pathways from all domains of life. MetaCyc contains 2,859 pathways from 3,185 different organisms [29]. It contains data about chemical compounds, reactions, enzymes, and metabolic pathways that have been experimentally validated and reported in the scientific literature. It covers both small molecule metabolism and macromolecular metabolism (e.g., protein modification). Figure 3 shows an example of a complete trehalose metabolic pathway involved in *Variovorax* sp. PAMC28711. MetaCyc is widely used in a variety of fields, including genome annotation, biochemistry, enzymology, metabolomics, genome and metagenome analysis, and metabolic engineering, duet to its exclusively experimentally determined results, intensive curation, comprehensive referencing, and user-friendly and highly integrated design. Although these two databases (KEGG and MetaCyc) have distinct features, both bioinformatics tools have certain drawbacks that should be considered when conducting research validation. It is important to note that different pathway databases have different pathway boundaries. The KEGG database favors complex metabolic maps that include all known reactions related to a general topic, regardless of whether they occur within the same species or even the same kingdom. UniPathway [30], on the other hand, designates every branching point as a linear subpathway border. MetaCyc lies in between these two databases [31].

## Conclusions

Before performing any kind of wet laboratory work, bioinformatics methods play a crucial role in predicting pathways. Online software has been proven to be useful in predicting research projects. Although commonly used online programs have good features, they have some limitations. In this study, we compared results of predicting trehalose metabolism pathways using two common databases. We found that both databases had some limitations as both databases showed enzymes missing for specific pathways. However, RAST annotation revealed that *Variovorax* sp. PAMC28711 possessed the enzyme maltooligosyl-trehalose synthase (TreY: EC 5.4.99.15) in the TreY/TreZ pathway for trehalose biosynthesis. Therefore, researchers should be aware of this when conducting preliminary screening employing bioinformatics tools. Many researchers are employing bioinformatics tools to predict their hypothesis before conducting any experiments. Our exploration of the trehalose metabolic pathway using two commonly used pathway databases demonstrated that bioinformatics tools might not provide accurate results. Thus, we need to evaluate databases before drawing definite conclusions.

## Data Availability

All data of this article can be found in the article itself.
